# Chronic pain in European adult populations: a systematic review of prevalence and associated clinical features

**DOI:** 10.1097/j.pain.0000000000003406

**Published:** 2024-10-08

**Authors:** Caroline Rometsch, Alexandra Martin, Florian Junne, Fiammetta Cosci

**Affiliations:** a Department of Experimental and Clinical Medicine, University of Florence, Florence, Italy; b School of Human and Social Sciences, University of Wuppertal, Wuppertal, Germany; c Department of Psychosomatic Medicine and Psychotherapy, University Hospital Magdeburg, Otto von Guericke University, Magdeburg, Germany; d Department of Health Sciences, University of Florence, Florence, Italy; e Department of Psychiatry and Neuropsychology, Maastricht University, Maastricht, the Netherlands

**Keywords:** Chronic pain, Persistent pain, Prevalence, Epidemiology, Clinical features

## Abstract

Chronic pain (CP) is a multifaceted and severely disabling persistent condition that affects individuals worldwide. A synthesis of epidemiological data is still lacking. The present systematic review aims at presenting point, period, and lifetime prevalence estimates of CP in the European adult population, as well as relevant features associated with it. Following PRISMA guidelines, a systematic search in PubMed, Web of Science, Embase, and Cochrane library was run from inception to May 2024, resulting in 39,832 hits. Among those, 132 full-text articles were assessed, and 23 studies comprising 862,013 participants with CP were included. The Joanna Biggs Institutes' Critical Appraisal Checklist for Studies Reporting Prevalence Data (JBI) was used for quality assessment. Findings showed a point prevalence ranging from 12% to 48%, a 6-month prevalence of 17.5% to 49.8%, a 12-month prevalence ranging from 8.1% to 44.6%, and a lifetime prevalence ranging from 12.7% to 33.7% independently from sex. Sociodemographic factors (eg, female sex, higher age, no partnership, lower education, unemployment) and clinical features (eg, medical diseases, mental disorders and burdens, healthcare utilization, functional impairment) were identified as being associated with CP. The variability in prevalence estimates can be reduced by applying consistent diagnostic criteria (eg, ICD-11) and by using standardized assessment tools (eg, scales, clinical interview). The high prevalence and the characteristics of the associated features support the need for adequate, multicomponent care paths to manage CP based on a comprehensive biopsychosocial model.

## Abstract 2

Supplemental Digital Content is Available in the Text.

## 1. Introduction

Chronic pain (CP) is an unpleasant sensory and emotional experience associated with, or resembling that associated with, actual or potential tissue damage.^59^ It causes disability^88^ and limitations in everyday life.^21^ It is associated with high direct medical costs^68^ and productivity losses^51^ because of reduced work ability and sick leaves.^66^

Chronic pain is subsumed under the category of “somatic symptom and related disorders” (SSDs) in the Diagnostic and Statistical Manual of mental disorders (DSM-5-TR).^4^ DSM-5 SSDs differ from previous editions thank to the inclusion, among diagnostic criteria, of psychological features, such as excessive thoughts, feelings, or behaviors related to somatic symptoms.^70^ However, the International Statistical Classification of Diseases and Related Health Problems (ICD-11) uses the diagnostic label “chronic primary pain,” which is identified when pain is persistent, longer than 3 months, associated with significant emotional distress and/or functional disability.^59^

Chronic pain is highly prevalent in the general population. The recently estimated point prevalence rates were 20% in the United States,^89^ 18% in developing countries,^74^ and 33% worldwide.^44^ Epidemiological data are essential for effectively planning healthcare paths and management of CP and for improving the quality of clinical care.^6^ Rometsch et al. (2024) provided a comprehensive overview of epidemiological data on functional disorders, reporting an overall point prevalence of 8.7% and a prevalence of CP of approximately 20% in Europe.^71^ However, the study lacks an in-depth analysis of associated features and specific prevalence rates of CP.

Sociodemographic (eg, age, sex, socioeconomic status), psychological (eg, anxiety, depression, beliefs about pain),^81^ and biological (eg, genetics, tissue damage, multimorbidity)^40,54,83^ variables have been associated with CP together with behavioral variables (eg, smoking, alcohol, physical activity). Some of them are modifiable factors (eg, sadness, lifestyle), others are not, because they occur in patients' biography^54^ (eg, injury, genetic components). A systematic overview of such variables is lacking, despite the wide knowledge that CP arises and persists because of a combination of different risk and protective ingredients.^23^ Identifying clinical pain–related factors (eg, pain location/severity), and also mental and physical disorders/comorbidities, might help in accurately diagnosing and treating CP, thus improving patient-centered healthcare paths and patient outcomes.^67^

In this framework, we aimed at conducting a systematic review and, when possible, a meta-analysis of epidemiological data of the adult general population across Europe regarding point, period, and lifetime prevalence of CP. Sociodemographic and clinical features associated with CP were identified, with particular attention to their prevalence, and their impact was discussed.

## 2. Methods

### 2.1. Eligibility criteria

Studies on prevalence of CP, published in peer-reviewed journals, in English language were included. Primary outcome was a prevalence estimate of CP, including point prevalence, 6- or 12-month prevalence, and lifetime prevalence.^60^ If not reported by the authors, point prevalence was calculated as percentage of diagnosed participants in the total sample ((ie, number of diagnosed participants/total sample number) x 100). The diagnosis of CP had to refer to DSM, ICD, or standardized criteria (eg, International Study of Pain). Studies with an observational design (eg, cross-sectional, longitudinal, cohort, case–control) on a representative (at least 500 subjects)^3,58^ European adult (≥18 years) general population were included. In case of reporting on children/adolescents and adults or both European and non-European countries, data were disaggregated, if possible. Inclusion criteria were followed by PICOs criteria.^53^ Special populations (eg, veterans, students), randomized controlled trials, and qualitative studies were excluded.

### 2.2. Information sources and search strategy

An expanded systematic search in PubMed, Web of Science, Embase, and the Cochrane library was run from inception to May 2024 to complement this existing study,^71^ particularly to identify associated features with the epidemiological data. The search terms were ((persistent pain) OR (chronic pain) OR (atypical pain)) combined with the Boolean “AND” operator with Prevalence* “OR” and “Epidemiol*” (see OSM for the detailed search strategy). A manual search of reference lists and a search of grey literature (eg, Opengrey) was conducted. Two reviewers (C.R. and F.C.) independently screened potential eligible articles and full texts. In case of disagreement, consent was achieved through discussion with a third reviewer (A.M. and F.J.).

The Preferred Reporting Items for Systematic Reviews and Meta-analysis (PRISMA) guidelines^65^ were followed. Endnote^41^ was used to remove duplicates. The online tool Rayyan^64^ was used for the systematic review process. The protocol was preregistered in Open Science Foundation^63^ (osf.io/8umya).

### 2.3. Data extraction and quality assessment

Relevant data were extracted through a standardized data extraction form which includes author and publication year, population, sample size, sex distribution, education, study design, region, diagnostic procedure, pain intensity, pain duration, and prevalence estimate. C.R. and F.C. independently verified the methodological quality of studies using the Joanna Biggs Institutes` Critical Appraisal Checklist for Studies Reporting Prevalence Data (JBI).^42^ This assessment tool proves study quality through 9 items exploring study participants, sample size, sample power, methods, measurement, statistical analysis, and response rate.^42^ Each item is scored on a 4-point Likert scale (“*yes*,” *“no*,” *“unclear*,” *“not applicable*”) with a maximum sum score of 9.^57^ A kappa coefficient of 0.94 showed a very good outcome for interrater reliability.^46^ Ratings are reported in the online supplementary material, http://links.lww.com/PAIN/C134.

### 2.4. Statistical analysis

Data analysis was performed using R Studio (version 4.3.0) with the R function *metaprop* from the R package meta (version 6.2.1). An overall point prevalence rate for CP was calculated using a generalized, linear, mixed-effects model (GLMM) that logit-transforms proportions.^37,49^ The study conducted by Wijnhoven et al.^86^ (2006) was excluded due to its reporting of prevalence rates exclusively for women and men separately.

The meta-analysis was performed only when at least 10 studies were available to ensure adequate power and robustness of the findings.^37^ A sensitivity analysis was performed to assess the robustness of the meta-analytic results, accordingly the studies of Macfarelane et al. (2015)^50^ and Brattberg et al. (1989)^11^ were excluded because older people of the general population were investigated. A subgroup analysis was performed focusing on studies with low and moderate risk of bias, as determined by the JBI rating. Between-study heterogeneity was examined by calculating τ^2^ with a maximum-likelihood estimator^84^ and using I^2^, Q-statistics, and prediction intervals.^39^ Results are presented with 95% confidence intervals (CI) assuming a Clopper–Pearson distribution and displayed using forest plots. To assess the association between the prevalence of CP and sociodemographic features, a meta-regression with the *metreg* function from the *metafor* package was used. The moderator variables included in the meta-regression were sex (percentage of females), marital status (percentage of individuals married or cohabiting), and employment status (percentage of employed individuals). To assess symmetry and publication bias, Egger test was conducted, and a funnel plot of logit-transformed proportions was created.

This study is part of the innovative training network ETUDE (Encompassing Training in Functional Disorders across Europe; https://etude-itn.eu/), which aims to enhance the understanding of mechanisms, diagnosis, treatment, and stigmatization of functional disorders.^72^

## 3. Results

### 3.1. Selection of articles and study characteristics

**Figure 1.**
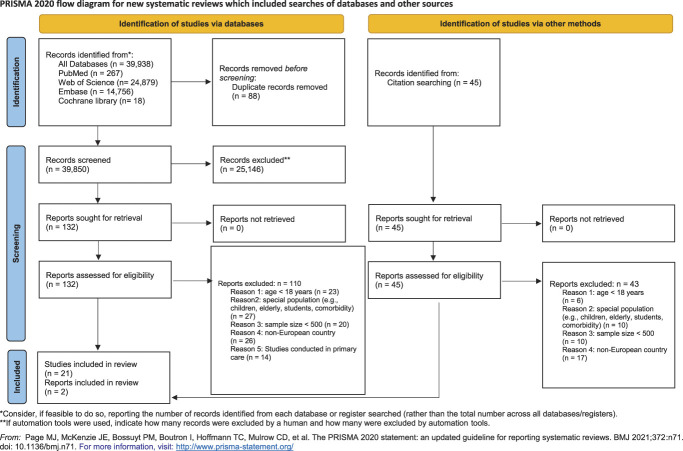
PRISMA flow diagram of study selection for the systematic review of chronic pain in European adult populations.

After removal of duplicates, 39,832 articles from databases and 45 from citation searching were screened for eligibility. Among them, 132 full-text articles were assessed. A total of 23 articles met the inclusion criteria (see PRISMA flow diagram Fig. 1) comprising 862,013 participants with CP and reporting on point (n = 16), and/or 6-month (n = 2) or 12-month (n = 8), and lifetime (n = 2) prevalence. Thus, meta-analysis and meta-regressions were conducted only for point prevalence of CP.

### 3.2. Prevalence rates of chronic pain

#### 3.2.1. Point prevalence estimates of chronic pain

In 16 studies, CP point prevalence ranged from 12%^12^ to 48%.^56^ The majority of these studies assessed the prevalence in specific body locations^5,10–13,15,22,35,43,50,73,86^ or regions (eg, head, neck and/or shoulder, back, abdomen, hip, arms and/or elbow, wrist and/or ankle, leg and/or knee),^8,15,19^ and only few independently from the location.^47,56,86^

#### 3.2.2. Period prevalence estimates of chronic pain

Eight studies reported on period prevalence. Two referred to 6-month prevalence, and 6- to 12-month prevalence. The 6-month prevalence was 17.5%^34^ and 49.8%.^2^ The 12-month prevalence rates reported were 8.1%,^32,45^ 23.9%,^7^ 28%^48^ up to 44.6%.^79^

#### 3.2.3. Lifetime prevalence rates of chronic pain

Lifetime prevalence was 12.7%^45^ and 33.7%.^34^

### 3.3. Features associated to point prevalence of chronic pain

Features associated to CP de facto referred to point prevalence and are described below (Tables 1 and 2).

**Table 1:** Main findings of sociodemographic features associated to the point prevalence of chronic pain.

Socio-demographic features	Details	References
Sex	CP more prevalent in females Females: headaches (22.7%) Males: lower extremities CP (24.1%) Severe pain in low socioeconomic status females Predictor of future CP	Chrubasik, Junck et al. 1998; Rustøen, Wahl et al. 2004; Wijnhoven, De Vet et al. 2006; Bouhassira, Lantéri-Minet et al. 2008; Björnsdóttir, Jónsson et al. 2013; Dueñas, Salazar et al. 2015; Macfarlane, Beasley et al. 2015; Mundal, Bjørngaard et al. 2016; Del Giorno, Frumento et al. 2017; Catala, Reig et al. 2002; Jablonska, Soares et al. 2006; Landmark, Dale et al. 2018.
Age	CP more prevalent at older age Peak: 45-64 y and over 65 y Younger (18-29 y): headaches Older (65+ years): lower extremities CP Knee and hip pain increase with age Predictor of future CP	Brattberg, Thorslund et al. 1989; Chrubasik, Junck et al. 1998; Catala, Reig et al. 2002; Rustøen, Wahl et al. 2004; Jablonska, Soares et al. 2006; Bouhassira, Lantéri-Minet et al. 2008; Azevedo, Costa-Pereira et al. 2012; Björnsdóttir, Jónsson et al. 2013; Dueñas, Salazar et al. 2015; Macfarlane, Beasley et al. 2015; Del Giorno, Frumento et al. 2017; Landmark, Dale et al. 2018.
Marital status	Higher risk in divorced and single individuals Contradictory results in widowhood Married individuals less at risk	Rustøen, Wahl et al. 2004; Jablonska, Soares et al. 2006; Björnsdóttir, Jónsson et al. 2013; Brattberg, Thorslund et al. 1989; Björnsdóttir, Jónsson et al. 2013; Jablonska, Soares et al. 2006.
Education and working activity	Lower education: predictor of CP Left education before 16: higher CP Higher education: fewer pain sites High prevalence in unemployed, disabled, sick, housewives, retired, farmers Predictor of future CP	Rustøen, Wahl et al. 2004; Jablonska, Soares et al. 2006; Azevedo, Costa-Pereira et al. 2012; Björnsdóttir, Jónsson et al. 2013; Macfarlane, Beasley et al. 2015; Mundal, Bjørngaard et al. 2016; Azevedo, Costa-Pereira et al. 2012; Macfarlane, Beasley et al. 2015; Rustøen, Wahl et al. 2004; Bouhassira, Lantéri-Minet et al. 2008; Catala, Reig et al. 2002; Landmark, Dale et al. 2018.
Social status and ethnicity	Higher CP in low income, financial/work strain, poor social support Family history of CP increases risk Asian and Black groups: higher CP Chinese: lower CP	Björnsdóttir, Jónsson et al. 2013; Macfarlane, Beasley et al. 2015; Bergman, Herrström et al. 2001; Jablonska, Soares et al. 2006; Macfarlane, Beasley et al. 2015; Macfarlane, Beasley et al. 2015.

CP, chronic pain.

**Table 2:** Main findings of clinical features associated to the point prevalence of chronic pain.

Clinical features	Details	References
Pain location, intensity, and duration	Highest prevalence: shoulder pain, headache Lowest prevalence: chest/stomach pain Female predominance: hip, wrist/hand pain 43% reported 1-2 pain sites, 56% reported 3+ pain sites Intensity assessed through metric or ordinal scales Women reported higher severity scores CP severity predicted pain interference with life activities Predictors of future CP: higher initial pain severity, widespread pain, long pain history	Svebak, Hagen et al. 2006; Fröhlich, Jacobi et al. 2006; Wijnhoven, De Vet et al. 2006; Mundal, Bjørngaard et al. 2016; Rustøen, Wahl et al. 2004; Breivik, Collett et al. 2006; Jablonska, Soares et al. 2006; Bouhassira, Lantéri-Minet et al. 2008; Azevedo, Costa-Pereira et al. 2012; Del Giorno, Frumento et al. 2017; Dueñas, Salazar et al. 2020; Wijnhoven, De Vet et al. 2006; Gunnarsdottir, Ward et al. 2010; Landmark, Dale et al. 2018.
Physical disease comorbidities	CP subjects had poorer physical health Common causes: strain injuries, arthritis, mechanical problems, disease, accidents Predictors: frequent illness, chronic disease 27% had a chronic disease or health problems Rheumatism: main cause of CP (18.3%) Associated with past injury, surgery, cancer Linked to obesity CP associated with physical mobility limitations	Björnsdóttir, Jónsson et al. 2013; Gunnarsdottir, Ward et al. 2010; Rustøen, Wahl et al. 2004; Catala, Reig et al. 2002; Chrubasik, Junck et al. 1998; Jablonska, Soares et al. 2006; Björnsdóttir, Jónsson et al. 2013.
Mental disorder comorbidities and mental burden	Poor mental health in CP subjects Reported depression (13%), sadness (32%), anxiety (29%) More depressive episodes correlated with higher CP prevalence Higher CP prevalence in those consulting for mental health issues Anxiety, depression, sleep problems, smoking, obesity: more pain sites Predictors of future CP: depression, catastrophizing pain, sleep problems Belief that mental disorders increase pain Factors for higher prevalence in females: biological, psychological, social	Björnsdóttir, Jónsson et al. 2013; Azevedo, Costa-Pereira et al. 2012; Dueñas, Salazar et al. 2015; Macfarlane, Beasley et al. 2015; Mundal, Bjørngaard et al. 2016; Landmark, Dale et al. 2018; Catala, Reig et al. 2002; Wijnhoven, De Vet et al. 2006.
Healthcare utilization	Two-thirds used multiple treatments Most used analgesics (93%): aspirin, paracetamol, acetaminophen, diclofenac, metamizol Females used medication more often Medication mainly recommended by healthcare professionals Self-medication higher in younger subjects Older subjects more often on treatment Predictors for healthcare consultation: increasing age, higher pain intensity, more pain sites, longer pain duration Nonpharmacological treatments used by one-fifth: heat application, massage, physical exercises, etc.	Chrubasik, Junck et al. 1998; Rustøen, Wahl et al. 2004; Gunnarsdottir, Ward et al. 2010; Catala, Reig et al. 2002; Del Giorno, Frumento et al. 2017; Chrubasik, Junck et al. 1998.
Functional impairment and life events	CP impaired life activities and personal health 27% of patients and 13.1% of performances impaired Common impairments: dressing, walking stairs, social activities, physical activities Low social support and poor family life More limitations in older subjects (65+ years) 25% on sick leave because of CP Work disability: 11% to 49% with financial issues 12% left or lost job because of CP Accidents as cause of CP in 11.2% of cases	Azevedo, Costa-Pereira et al. 2012; Björnsdóttir, Jónsson et al. 2013; Dueñas, Salazar et al. 2015; Gunnarsdottir, Ward et al. 2010; Catala, Reig et al. 2002; Brattberg, Thorslund et al. 1989; Dueñas, Salazar et al. 2015; Jablonska, Soares et al. 2006; Azevedo, Costa-Pereira et al. 2012; Rustøen, Wahl et al. 2004.

CP, chronic pain.

#### 3.3.1. Sociodemographic features

##### 3.3.1.1. Sex

Chronic pain was found to be more prevalent in women,^8,10,15,19,22,50,73,86^ and female sex predicted CP.^19,47^ Women had CP mostly from headaches (22.7%), and men had CP in the lower extremities (24.1%).^13^ In case of low socioeconomic status, women experienced more severe and disabling pain.^43^

##### 3.3.1.2. Age

Chronic pain was more prevalent at older age,^5,8,10,11,13,15,19,22,43,50,73^ particularly severe pain was associated with a higher age.^19^ A peak was described between 45 and 64 years of age^11^ and older than 65 years.^13^ Younger subjects (18-29 years of age) suffered more often from headaches, and those older than 65 years suffered most often from CP in lower extremities.^13^ In addition, the prevalence of pain in the knee and hip increased with age.^50^ Higher age was a predictors of future CP.^47^

##### 3.3.1.3. Marital status

Divorced and single participants^8,43,73^ were at higher risk of developing CP than those married.^11^ In case of widowhood, results are contradictory.^8,43^

##### 3.3.1.4. Education and working activity

Lower education was a predictor of CP.^5,8,43,73^ Those who left education before 16 years of age reported higher CP compared with those who stayed in education until at least 17 years.^50^ A lower education level was a significant predictor of future CP.^47^ Highest prevalence rates were found in case of unemployment,^5,50^ in work disabled or sick people,^50^ in housewives, in retired people,^5,10,73^ and in farmers.^8,13^

##### 3.3.1.5. Social status, family history, and ethnicity

Chronic pain prevalence was higher in subjects with lower income,^8,50^ financial or work strain, and poor social support.^7,43,50^ Having a family history of CP was associated with a higher prevalence of CP.^50^ Asian and Black ethnic groups reported higher prevalence of pain compared to White participants.^50^ Chinese individuals had a lower prevalence of CP compared with other ethnic groups.^50^

#### 3.3.2. Clinical features

##### 3.3.2.1. Pain location, intensity, and duration

The highest point prevalence was found for shoulder pain^79^ and headache,^32^ and the lowest for chest/stomach pain.^79^ The highest female predominance was observed for hip and wrist/hand pain.^86^ Pain intensity and duration differed among the studies, also regarding the assessing methods. Intensity was assessed through metric^10,12,43,73^ or ordinal scales.^5,19,21^ Chronicity was described using different cutoffs of duration of pain^32^ (Table 2). Women reported higher severity scores than men, particularly for elbow, wrist/hand, and foot pain.^86^ Chronic pain severity significantly predicted pain interference with life activities.^35^ Pain interference mediated the effects of CP severity on positive affect, negative affect, and global quality of life.^35^ Factors that significantly predicted the development of future moderate to severe CP included higher initial pain severity and widespread pain.^47^ The likelihood of developing and recovering from CP was strongly influenced by the duration and history of previous pain and was more likely to continue in individuals with a long history of pain^47^ (Table 3).

**Table 3:** Pain intensity and duration in European adults according to the studies included in the systematic review.

	Author	Country	Age	Pain intensity	Pain duration
1.	Brattberg et al. (1989)^11^	Sweden	18-84 Mean age: N/A Age groups: 18-44: 126/368 45-64: 138/276 65-84: 66/183	3 of 4 respondents reported pain intensity as ‘like being stiff after exercise’ or worse, regardless of pain localization	N/A
2.	Andersson et al. (1994)^2^	Sweden	25-74 25-34 y: 16.4% 35-44 y: 26.2% 45-54 y: 20.3% 55-64 y: 19.4% 65-74 y: 17.7%	Grade 1 (weak pain): 11.6% Grade 2 (mild pain): 22.6% Grade 3 (moderate pain): 33.1% Grade 4 (severe pain): 12.8% Grade 5 (intense pain): 19.8%	50% of the sample: 3 mo or longer 49% of the sample: 8 mo or longer
3.	Chrubasik et al. (1998)^15^	Germany	18-80 Mean age: N/A	Differentiation in mild, severe, intolerable pain intensity according to the specialization of care giver were consulted	N/A
4.	Bergman et al^7^	Sweden	Mean age: 46.5 y	N/A	N/A
5.	Catala et al. (2002) (Catala et al., 2002)	Spain	18-29 y: 20.13% 30-44 y: 25.07% 45-64 y: 33.72% 65+ years: 41.75%	N/A	N/A
6.	Grabe et al. (2003) (Grabe et al., 2003)	Germany	Mean age of onset: 30.2 ± 13.2 y in males, 23.3 ± 12.6 in females.	N/A	9.5 ± 9.6 y in males, 13.8 ± 12.1 in females.
7.	Rustøen et al. 2004^73^	Norway	19-81, Mean age: 45.2 y	Mean score of 5.79 (SD 2.2) where 0 = “not at all bothered” and 10 = “unbearably bothered by their pain”	Mean duration of pain: 13.2 y (SD = 12.3, range 3 mo to 58 y) 34% reported chronic pain from 3 mo to 5 y, 22% from 6 to 10 y, 23.4% from 11 to 20 y 21% reported being in pain for over 20 y
8.	Jacobi et al. (2004)^45^	Germany	18-79, Mean age: N/A	Not reported	Reports on both 12-mo and lifetime prevalence, no further investigation on duration
9.	Breivik et al. (2006)^12^	Spain, Ireland, UK, France, Switzerland, Denmark, Germany, The Netherlands, Sweden, Finland, Austria, Belgium, Italy, Poland, Norway	≥18, Mean age: 49.9	66% reported a moderate pain, score of 5-7 on a 1-10-point numeric rating scale (NRS) 34% reported a severe pain, score of 8-10 on a 1-10-point NRS scale	N/A
10.	Fröhlich et al. (2006)^32^	Germany	18-65, Mean age: N/A	Not reported	N/A
11.	Jablonska et al. (2006)^43^	Sweden	18-64, Mean age: 41.6	Mean pain intensity was 6 (NRS 0-10) (SD = 2.4)	N/A
12.	Svebak et al. (2006)^79^	Norway	≥20, N/A	N/A	Average duration: 10.3 y
13.	Wijnhoven et al. (2006)^86^	The Netherlands	18-64, N/A	Score 1 (minor pain): males: 11.7%; females: 11.5% Score 2: males: 22.6%; females: 22.7% Score 3: males: 35.2%; females: 31.0% Score 4: males: 14.5%; females: 11.2% Score 5 (intense pain): males: 16.0%; females: 23.6%	N/A
14.	Bouhassira et al. (2008)^10^	France	≥18, mean age: N/Age (y) <25: 9.5% (2245) 25-34: 17.9% (4232) 35-49: 25.7% (6106) 50-64: 22.1 (5240) 65-74: 15.7 (3725) ≥75: 9.1 (2164)	Mean pain intensity: 4.5 ± 2.1 (NRS 0-10). Mild: 33.9% (ie, mean intensity = 1-3) Moderate: 46.5% (ie, mean intensity = 4-6) Severe: 16.1% (ie, mean intensity = 7-10)	<6 mo: 9.8% of the sample 6-12 mo: 15.3% of the sample 12-36 mo: 25.9% of the sample >36 mo: 48.7% of the sample
15.	Gunnarsdottir et al. (2010)^35^	Iceland	≥18, mean age: 44.94 y	Worst pain in the past week: mean 4.85 (SD 2.17) on a scale from 0 (no pain) to 10 (pain as bad as I can imagine) Least pain in the past week: mean 1.92 (SD 1.86) Average pain in the past week: mean 3.35 (SD 1.93) Current pain: mean 2.70 (SD 2.27) Composite pain severity score: mean 3.21 (SD 1.73)	Median duration of pain: 3 y
16.	Landmark et al. (2012)^48^	Norway	≥20 20-44: 1356 (28%), 45-64: 2265 (48%), 65+: 1161 (24%)	N/A	N/A
17.	Azevedo et al. (2012)^5^	Portugal	≥18, Mean age: N/A	From moderate to severe intensity: 68%	N/A
18.	Björnsdóttir et al. (2013)^8^	Iceland	18-79, N/A	N/A	N/A
19.	Dueñas et al. (2015)^22^	Spain	≥18, Mean age: 56.5 18-44: 943 (48%) 45-64: 600 (31%) 65+: 414 (21%)	Moderate: 43.4% Severe: 35%	Mean duration: 10 y
20.	McFarlane et al. (2015) (Macfarlane, Beasley, Smith, Jones, and Macfarlane, 2015)	UK	N/A	Reporting of chronic pain (each of 100%) 40-44 y: 39.6% 45-49 y: 42.3% 50-54 y: 43.6% 55-59 y: 43.8% 60-64 y: 43.8% 65-69 y: 45.7%	N/A
21.	Mundal et al. (2016)^56^	Norway	≥20, mean age: 44.94 y, (SD = 17.2)	Worst pain: mean 4.85 (SD 2.17) Least pain: mean 1.92 (SD 1.86) Average pain: mean 3.35 (SD 1.93) Current pain: mean 2.70 (SD 2.27) Composite pain severity score: mean 3.21 (SD 1.73)	Median pain duration: 3 y
22.	Del Giorno et al. (2017)^19^	Italy	≥18, median age: 58 y	Light: 4.4% Moderate: 42.8% Severe: 51.5%	N/A
23.	Landmark et al. (2018)^47^	Norway	≥20, 20-44 y: 28.6% 45-64 y: 47.7% 65+ years: 24.3%	Mild pain: 19.1% Moderate to severe pain: 27.7%	N/A

##### 3.3.2.2. Physical disease comorbidities

Subjects with CP had more often a poor physical health status than subjects without CP.^8^ Common causes of CP included strain injuries (work/sports), arthritis, mechanical problems, disease (eg, arthritis, heart, or kidney diseases), and accidents.^35^ Being frequently ill or having a chronic disease were predictors of CP.^73^ Twenty-seven percent of subjects with CP suffered from a chronic disease or had health problems, such as gastrointestinal diseases (8.9%), musculoskeletal problems (8.8%), osteoarthritis (7.5%), and asthma (7.1%).^73^ Rheumatism was a main cause for CP (18.3% of the sample).^13^ Affected subjects reported past injury (20.3%), surgery (7.9%), or cancer (2%).^73^ Chronic pain was associated with obesity,^8,15^ with 71% having a BMI of >25 kg/m^2^ when pain was frequent compared with 29% with a BMI of <25 kg/m^2^.^43^ Chronic pain was associated with deficient energy, muscular discomfort, physical mobility limitations, lifting grossness, dumbing stairs, and stooping.^8^

##### 3.3.2.3. Comorbidities with mental disorders and mental burden

Subjects with CP had a poor mental health.^8^ They reported depression (13%),^5^ sadness (32%), or anxiety (29%).^22^ Increased number of depressive episodes correlated with higher prevalence of CP.^50^ Subjects who consulted a general practitioner for anxiety, tension, or depression had a higher prevalence of CP.^50^ Depression, catastrophizing pain, and sleep problems predicted the development of moderate to severe CP.^47^ Subjects with CP believed that mental disorders increase pain.^13^

##### 3.3.2.4. Healthcare utilization

Two-third of CP subjects used more than only one treatment.^15^ Most subjects with CP (93%) used analgesics,^35,73^ namely, aspirin (54.3%),^13^ paracetamol (22%),^19^ acetaminophen (15%),^13^ diclofenac (5%),^13^ and metamizol (4.8%).^13^ They also used glucocorticoids (7%), pregabalin (2%), and gabapentin (1%).^19^

Women used medication more often than men.^19^ Medication was mostly recommended by a physician or nurse (66%). Self-medication was reported by 29% of cases, mainly by younger subjects (44.4% of subjects < 29 years vs 19.2% of subjects > 65 years),^13^ who also searched for medical help more frequently than older ones.^13^ Subjects at older age were found to be more often on treatment.^19^

Predictors for healthcare consultation and number of treatments (sought by the patients) were increasing age, higher pain intensity, higher number of painful sites, and longer pain duration.^15^

One-fifth of affected subjects used nonpharmacological treatments: heath application, massage, mud pads, physical exercises, electrical therapy, spa treatments, relaxation, acupuncture, herbal medicine, surgeries, TENS, and nerve blocks with a self-referred treatment success.^15,35,73^

##### 3.3.2.5. Functional impairment and life events

Chronic pain leads to impaired life activities^5,8,22^ and to a worse personal health.^35^ In 27% of subjects, performances,^13^ such as dressing (6.4%), walking stairs (129%), and social activities (26%),^11^ were impaired. In addition, physical activities,^8^ such as lifting weight, bending, kneeling, crouching, going up or down a flight of steps, washing, bathing, or combing one's hair, were diminished.^22^ A possible consequence was a low social support^43^ and a poor family life.^5,22^ Limitations in everyday activities were found to be more common in subjects older than 65 years (35.8%) than in younger ones (<29 years, 21.1%).^13^ Sick leave is asked and obtained by one-quarter of the subjects with CP in a year.^22^ Subjects with CP and a lower education took less sick leave than those with higher academic achievements.^22^ Chronic pain led to work disability,^5,43^ from 11%^13^ to 49% of individuals^5^ with financial issues as an implication.^43^ Approximately 12% of the subjects with CP had left or lost their job because of CP.^22^

Having an accident, as life event, was interpreted as a cause of CP in 11.2% of cases.^73^

### 3.4. Features associated to 6- and 12-month prevalence of chronic pain

#### 3.4.1. Sociodemographic features

##### 3.4.1.1. Sex and age

Chronic pain was found to be more prevalent in women^2,32,45,48,56,79^ and at older age,^2,7,48,79^ in particular between 55 and 59 years.^2,7^ The prevalence of chronic pain increased with age, with a peak in the 50 seconds for women and in the 60 seconds for men.^79^ Age of onset of CP was 19 years.^45^ Women reported the highest pain intensity.^2^ Chronic pain prevalence in neck, shoulder, forearm, hip, and hand was higher in women than in men.^2^

##### 3.4.1.2. Social status, family history, and working

A predictor of CP was a lower education.^48^ Higher education (university/college) was associated with fewer pain sites.^56^ Highest prevalence rates were described in case of unemployment,^32^ manual workers,^7^ and blue collars.^2^ A positive family history of CP was associated with a higher prevalence of CP.^7^ The prevalence of sick leave and reduced capacity at work/leisure time increased with the number of pain locations.^79^ One in 4 participants reported taking sick leave in the past year because of CP.^79^

#### 3.4.2. Clinical features

##### 3.4.2.1. Pain location and duration

Subjects suffered most often from headaches (12.7%; females 17% vs males 8%), abdominal pain (8.3%; females 17% vs males 8%), and back pain (8.5%).^32^ Highest prevalence rates were found for chronic shoulder pain with 20.3% in women and 15.7% in men, lowest prevalence rates were found in the chest/stomach (5.1% in women vs 3.7% in men).^79^ Approximately 43% of patients with CP reported 1 to 2 pain sites, whereas 56% reported 3 or more pain sites.^56^ The prevalence of CP increased in lower extremities at 55 to 59 years, and the prevalence of CP in neck and shoulders increased at 45 to 64 years.^2^ The average duration of CP was 10.3 years^79^ (Table 2).

##### 3.4.2.2. Physical disease or mental disorder comorbidities

Twenty-two percent of CP subjects had at least one additional comorbid physical disease.^45^ Comorbidity with mental disorder (eg, major depressive disorder, dysthymia, bipolar disorder, anxiety disorders, obsessive–compulsive disorder) was observed in 28% of subjects.^32^ Generalized anxiety disorder was found in 7.1% of patients with CP, whereas dysthymia was observed in 16.7%.^32^ Women had higher prevalence rates of mood (16% vs 7%) and anxiety disorders (20% vs 7%) than men who had a higher prevalence of substance use disorders (6% vs 2%).^32^ Higher prevalence of CP was observed in current or former smokers.^7^ Subjects with anxiety and/or depression, sleep problems, smoking, and obesity had an increased number of pain sites, with the strongest associations observed among those with anxiety and depression combined, sleep problems together with obesity.^56^

##### 3.4.2.3. Functional impairment

Patients with CP had a poorer quality of life (measured by the SF-36) than those without CP.^32^ They had on average 29.7 (*SD* = 34.8) disability days per year.^32^

### 3.5. Features associated to lifetime prevalence of chronic pain

Onset of CP was earlier in women (on average at 23 years of age) than in men (on average at 30 years of age).^34^ Men more often retired earlier because of CP than women and showed lower income.^34^ Women suffered longer from CP than men (14 vs 9.5 years).^34^ Most subjects (98%) contacted their medical doctor because of pain^34^; in 56% of the cases, a mental disorder was diagnosed, and in 20%, an associated physical problem was observed.^34^

### 3.6. Meta-analysis of point prevalence of chronic pain

**Figure 2.**
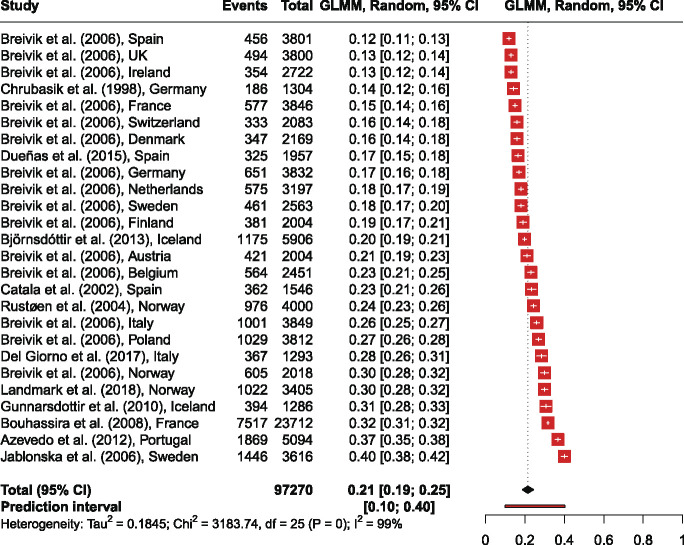
Forest plot of the studies on point prevalence of chronic pain.

After conducting a sensitivity analysis including 28 studies and 601,604 observations, an overall point prevalence of 22.62% [CI 19.50% to 26.07%] was found with τ^2^ = 0.23, I^2^ = 99.8%. After excluding studies with participants with higher age, which applies to the studies of Macfarelane et al. (2015)^50^ and Brattberg et al. (1989),^11^ the meta-analysis with 26 studies and 97,270 observations resulted in an overall point prevalence of 21.45% [CI from 18.66% to 24.54%], τ^2^ = 0.18, I^2^ = 99.2% (Fig. 2).

### 3.7. Meta-regression on sociodemographic features and chronic pain prevalence

The mixed-effects meta-regression model investigating the influence of sex on the point prevalence of CP, which included 26 studies, showed a nonsignificant result (F_(1,24)_ = 3.25, *P* = 0.084, see Table 4). The analysis revealed significant residual heterogeneity (tau^2^= 0.164, I^2^ = 99.00%). Similarly, analysis on the marital status, including 6 studies, showed a nonsignificant result (F_(1,4)_ = 7.26, *P* = 0.055, see Table 5) with significant residual heterogeneity (tau^2^= 0.049, I^2^= 96.89%).

**Table 4:** Meta-regression analysis on the associations of sex with the point prevalence of chronic pain.

Variable	Coefficient	SE	t-value	95% CI lower	95% CI upper	*P*
Intercept	−2.148	0.479	−4.489	−3.135	−1.160	0.0002
Sex (females)	0.015	0.008	1.802	−0.002	0.032	0.0841

95% CI, 95% confidence interval with lower and upper bounds; SE, standard error.

**Table 5:** Meta-regression analysis on the associations of sex, marital status, and occupational status with the point prevalence of chronic pain.

Variable	Coefficient	SE	t-value	95% CI lower	95% CI upper	*P*
Intercept	−2.3129	0.5437	−4.2538	−3.8226	−0.8033	0.0131
Marital status (married/partnership)	0.0241	0.009	2.6936	−0.0007	0.049	0.0545

95% CI, 95% confidence interval with lower and upper bounds; SE, standard error.

### 3.8. Publication bias

**Figure 3.**
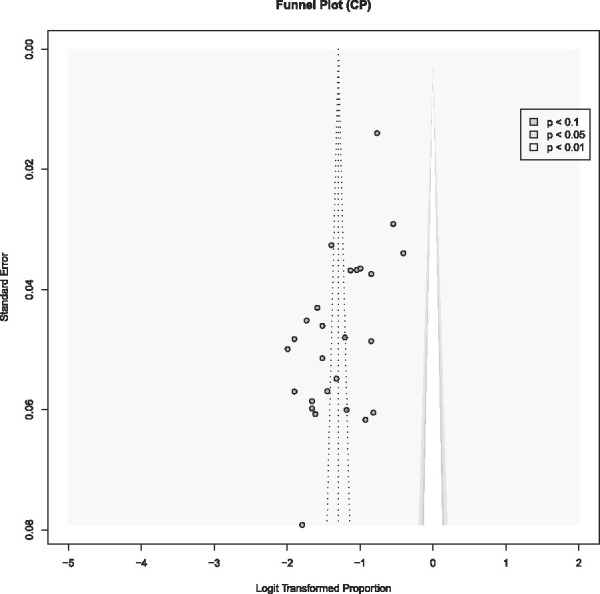
Funnel plot of the studies on point prevalence of chronic pain.

The Eggers test showed significant asymmetry of the included studies (t(24) = −4.35, *P* < 0.001), which was confirmed by and the funnel plot (Fig. 3), indicating the presence of publication bias or small-study effects.

### 3.9. Risk of bias

For the meta-analysis on point prevalence, 5 studies demonstrated a low risk of bias, revealing an overall point prevalence of 26.28% (95% CI: 17.80%-36.98%). However, 21 studies exhibited a moderate risk of bias, with an overall point prevalence of 20.40% (95% CI: 17.50%-23.63%). The subgroup analysis indicated a nonsignificant effect between the groups (Q(1) = 2.70, *P* = 0.10).

## 4. Discussion

The present systematic review showed a high prevalence of CP in the adult population across Europe and presented relevant features associated to CP. Point prevalence of CP was 12% to 48%, 6-month prevalence was 17.5% to 49.8%, 12-month prevalence was 8.1% to 44.6%, and lifetime prevalence was 12.7% to 33.7%. The overall point prevalence was 21.45% [CI from 18.66% to 24.54%]. The primary studies included in the systematic review suggest the impact of various sociodemographic (eg, female sex, higher age, not having a partner, lower education, lack of working activity) and clinical (eg, physical disease, mental disorders and burden, medication utilization, and functional impairment) variables on the prevalence of CP.

The point prevalence shows a relatively wide range, which is in line with systematic reviews conducted on data collected in the US population (point prevalence 11%-40%)^17^ or in developing countries (point prevalence 13%-51%).^74^ Systematic reviews conducted in Brazil (point prevalence 23%-76%),^1^ Asia (point prevalence 7%-61%),^90^ or worldwide (point prevalence 8.7%-64%)^78^ showed even wider ranges. Systematic reviews of period and lifetime prevalence, considering CP as a whole, are missing, although some refer to specific diagnoses. For instance, systematic reviews on chronic low back pain showed a 12-month prevalence between 22% and 65% and a lifetime prevalence between 11% and 84%^85^; those on chronic neck pain showed a 12-month prevalence between 16.7% and 75.1% and a lifetime prevalence between 14.2% and 71%.^30^

Heterogeneity in point prevalence might arise from the diagnostic criteria applied. Different nosographic systems were used, most often those proposed by the International Association for the Study of Pain (IASP)^5,8,11,13,22,73^ and also the DSM.^32,34,45^ Some studies referred to standardized criteria using different cutoffs for CP duration, ie, 3 months^10,43^ or 6 months.^15,48^ A commonly used taxonomy seems to be essential to limit such type of heterogeneity, which might also allow to run meta-analyses of data with heterogeneity. The ICD-11, being a World Health Organization tool, might help in overcoming such limitation.^59^ In addition, it showed to capture the complexity of CP including the distinction between primary and secondary^61,80^ and referring to significant emotional distress or functional disability (eg, everyday life activities or social participation),^59^ thus to psychosocial aspects of the illness. Point prevalence might be heterogeneous also because of the assessment tools applied. Prevalence was lower when using an interview^5,12,13,19,22,32,34,45^ than a questionnaire.^8,10,11,15,43,48,73,78^

Together with the application of uniform diagnostic criteria (such as ICD-11), the employment of standardized assessment tools, including clinical interviews, might help in reducing such variability.

Finally, the prevalence was described only for some EU countries (ie, Austria, Belgium, Finland, France, Germany, Iceland, Ireland, Italy, Norway, Poland, Portugal, Spain, Sweden, Switzerland, The Netherlands, United Kingdom), which have shown an historical attention toward CP and psychosomatic conditions in general^91,92^ and which have addressed more financial sources to the care of CP (eg, Denmark, Germany, Spain).^62^ Although this study aimed to systematically analyze the prevalence for European countries, missing data were observed (eg, Bulgaria, Croatia, Cyprus, Czech Republic, Denmark, Estonia, Greece, Hungary, Latvia, Lithuania, Luxembourg, Malta, Romania, Slovakia, and Slovenia).

Literature on features of CP refers mainly to point prevalence, and the sparse data on period or lifetime prevalence do not add much. Concerning sociodemographic features, being women, having older age, being single, having a lower education, lacking an employment, and presenting lower socioeconomic status (SES) were associated to CP,^54^ although those variables (sex, marital status, occupational status) were not statistically significant in the meta-regression to predict the prevalence rate. The latter may be primarily because of the small number of studies included in the analysis. Nevertheless, these features are relevant in terms of care demand. Women and older patients with CP often do not ask for an adequate assessment and are less likely to receive a multimodal therapy or psychotherapy.^52,69^ Patients' motivation to search for medical help is strongly dependent on the levels of pain-related disability,^9^ and overall, patients with lower SES showed a higher healthcare utilization and higher rates of comorbidities.^20^ However, patients with low SES faced more likely barriers to care.^77^ This underlines the importance of offering targeted treatment access for CP patients in a public, well-organized, and patient-centered health system environment.^55^

Clinical features are overall consistent with the worldwide literature on CP.^54^ Physical or mental co-occurrences are highly associated to CP, which is mainly treated through polypharmacotherapy,^31^ although approximately one-third of individuals self-prescribe medications.^75^ This may contribute to a medication misuse in an era in which pain has been largely treated with opioids, which have implied major health problems in terms of opioid dependence epidemic and mortality.^76^

The impact of CP is also evident in terms of individual functioning and sick leaves, which are observed in about one-fourth of patients^66^ and stresses the need of patients' self-management to overcome unhealthy lifestyles.^27^

Under the light of the wide knowledge, we have on the interplay between cellular, tissue, neural, organic, interpersonal, psychological, social, and environmental factors,^25^ the present results emphasize the need of specific tools and pain-targeting care paths to assess and treat chronic pain comprehensively, thus including the psychosocial components.^18^ A multiparametric assessment, for which Feinstein (1983) introduction of *clinimetrics* is an important orientation, is recommended. It comprises, among the others, the psychosocial impact of ailments, the restrictions in physical activity, the relation to the patients` family, joys, and sorrows^29^ through the use of macro-analysis, a clinimetric tool that identifies patient's main problematic areas, independently from the fulfilling of diagnostic criteria, and establishes a relationship among them to guide the clinician in the choice of what to treat first.^24,28^ It may also comprise the use of Diagnostic Criteria for Psychosomatic Research^26^ as a complementary diagnostic system able to catch psychosocial manifestations, such as demoralization and irritable mood, which do not find room in the customary taxonomy. Assessing demoralization is of priority in CP patients because it captures a psychological distress, which occurs commonly.^82,87^

The systematic synthesis of prevalence estimates, together with the meta-analysis of point prevalence data, also indicates that a significant portion of the general population is affected by CP, highlighting the need for an adequate healthcare system offering pain-targeting care based on the biopsychosocial model. National guidelines recommend therapies to be patient-centered, flexible, and multimodal, ideally in an interdisciplinary treatment environment^33^ with a stepped-care approach.^38^ Comprehensive pain-targeting multimodal treatment includes restorative therapies (eg, physical or activation therapy), pharmacotherapy, procedural interventions, psychological therapy, such as behavioral treatments, and complementary and integrative therapies (eg, acupuncture, dietary supplement).^16^ Ideally, healthcare systems should have multidisciplinary pain clinics.^14^ Their development and implementation, supported by properly trained pain specialists, are essential steps in addressing CP management in the healthcare systems.^36^

The primary limitation of this study is the relatively limited number of studies, selected for robustness, which could lead to potential overestimation or underestimation of CP prevalence and might result in the omission of crucial CP characteristics. The effects of the meta-regression were not statistically significant, which might be solved by including larger data sets in future studies. The analysis primarily evaluated the influence of sample characteristics on prevalence rates to assess their robustness. Additional factors, such as distress or impairment, may also be pertinent. In addition, individual patient data meta-analyses might be more appropriate to identify the association between the variables of interest. In conclusion, systematically reviewing individual study results, as conducted in this work, is crucial for understanding the epidemiological data. The Egger test showed a significant result. Smaller studies tend to have higher prevalence estimates, which could influence the overall pooled prevalence. This might true for the studies of Del Giorno et al. (2017),^19^ Gunnarsdottir et al. (2010),^35^ and Chrubasik et al. (1998).^15^ The risk of bias analysis did not show any significant difference between high or moderate quality of included studies. Meta-analyses and moderator analyses on the association of the features were not possible due to the heterogeneity of the data and might be focus of future research.

Overall, this study shows that chronic pain is a complex health issue in need of being investigated, assessed, treated under the light of psychosomatic medicine. This is the only clinical discipline, which guarantees multidisciplinary, interdisciplinary, individualized approaches and strategies. Psychosomatic medicine is more and more adequate in dealing with symptoms and problems, which cut across organ system subdivisions and require a comprehensive evaluation.^26^ The philosophy is, indeed, based on Whipple “all-under-one-roof” principle, which he felt would facilitate collegiality, cooperative education, research, and care and, in turn, make the barriers between departments and disciplines “freely permeable” (John Romano personal communication to Jules Cohen, 1985-94).

## Conflict of interest statement

The authors have no conflicts of interest to declare.

## Supplementary Material

## Supplemental digital content

Supplemental digital content associated with this article can be found online at http://links.lww.com/PAIN/C134.
