# Supplementary material for: Chronic pain in European adult populations: a systematic review of prevalence and associated clinical features
Source: Pain. 2024 Oct 8;166(4):719–31. doi: 10.1097/j.pain.0000000000003406 (PMC11921450; doi:10.1097/j.pain.0000000000003406)
Supplement: Supplementary file 1 [file jop-166-719-s001.pdf]

Online supplementary material

3.2.2 Studies reporting on chronic pain which were included in the systematic review of the literature

	Author	Year	Country	Sample size	Sex distribution	Age	Education	Participant recruitment	Region	Study design	Diagnostic criteria	Diagnostic instrument	Clinical interview	Type of prevalence measure	Prevalence estimate	JBI
**1.**	Brattberg et al. [6]	1989	Sweden	1,009	Males: 45.9%, Females: 54.1%	18–84	N/A	Postal survey, Registry, Random sample	Urban	Cross-sectional	IASP	Self-developed	No	Point prevalence	40%	6
**2.**	Andersson et al. [1]	1994	Sweden	1,086	Males 50,8%, Females 49,2%	25–74		Population register	Rural	Cross-sectional	Defintion: 3 or 6 month	Self-developed	No	6-month prevalence	49.8%	8
**3.**	Chrubasik et al. [9]	1998	Germany	1,304	Males: 45.9%, Females: 54.1%	18–80	Years of Education:<br><br>Women:	Postal survey, Registry, Random sample	Diverse	Cross-sectional	Definition: 6 months	Self-developed	No	Point prevalence	14.3% (12.4-16.2)	6

	Author	Year	Country	Sample size	Sex distribution	Age	Education	Participant recruitment	Region	Study design	Diagnostic criteria	Diagnostic instrument	Clinical interview	Type of prevalence measure	Prevalence estimate	JBI
							≤10: 13.1%<br>11–13: 44.4%<br>≥14: 42.5%<br>Men:<br><br>≤10: 10.0%<br>11–13: 55.3%<br>≥14: 34.7%									
**4.**	Bergman et al. [3]	2001	Sweden	2,425	Males 46.8%<br>Females 53.2%	20–74	N/A	Population register	Diverse	Cross-sectional	Definition: 3 month	SF-36	No	12-month Prevalence	23.9%	8

	Author	Year	Country	Sample size	Sex distribution	Age	Education	Participant recruitment	Region	Study design	Diagnostic criteria	Diagnostic instrument	Clinical interview	Type of prevalence measure	Prevalence estimate	JBI
**5.**	Catala et al. [8]	2002	Spain	1,546	Males: 20.9%, Females: 37.6%	18–95	N/A	Telephone survey	Diverse	Cross-sectional	IASP	Self-developed	Yes	Point prevalence	23.4%	6
**6.**	Grabe et al. [13]	2003	Germany	4,075	Males: 46.3%, Females: 53.7%	18–64	Years of Education:<br><br>Males:<br><br>9 years: 54.5%<br>10 years: 19.4%<br>12–13 years: 21.8%<br>Other: 4.2%<br>Females:	Random sample from registration office files	Diverse	Cross-sectional	DSM-III-R criteria	M-CIDI	Yes	6-month, lifetime prevalence	17.3%, 33.7%	8

	Author	Year	Country	Sample size	Sex distribution	Age	Education	Participant recruitment	Region	Study design	Diagnostic criteria	Diagnostic instrument	Clinical interview	Type of prevalence measure	Prevalence estimate	JBI
							9 years: 46.7%<br>10 years: 35.0%<br>12–13 years: 15.0%<br>Other: 3.3%									
**7.**	Rustøen et al. [21]	2004	Norway	4,000	Males: 48%, Females: 52%	19–81	Educational Status:<br><br>Primary school: 19%<br>Secondary school: 44%	National Register	Diverse	Cross-sectional	IASP	Self-developed, Brief Pain Inventory	No	Point prevalence	24.4%	7

	Author	Year	Country	Sample size	Sex distribution	Age	Education	Participant recruitment	Region	Study design	Diagnostic criteria	Diagnostic instrument	Clinical interview	Type of prevalence measure	Prevalence estimate	JBI
							University <4 years: 20% University >4 years: 17%									
**8.**	Jacobi et al. [16]	2004	Germany	7,124	Males: 50.3%, Females: 49.7%	18 – 65	Educational Distribution (Social Class Index): Low: 19.1% Medium: 57.6%	Population registries, Stratified random sample	Diverse	Cross-sectional	DSM-IV	M-CIDI	Yes	12-month prevalence, Lifetime prevalence	8.1%, 12.7%	8

	Author	Year	Country	Sample size	Sex distribution	Age	Education	Participant recruitment	Region	Study design	Diagnostic criteria	Diagnostic instrument	Clinical interview	Type of prevalence measure	Prevalence estimate	JBI
							High: 23.3%									
**9.**	Breivik et al. [7]	2006	Spain, Ireland, UK, France, Switzerland, Denmark, Germany, Netherlands, Sweden, Finland, Austria, Belgium, Italy,	Spain: 3,801, Ireland: 2,722, UK: 3,800, France: 3,846, Switzerland: 2,083, Denmark: 2,169, Germany: 3,832, Netherlands: 3,197,	Males: 44%, Females: 56%	≥18	N/A	Telephone survey	Diverse	Cross-sectional	Definition: pain duration more than 6 months	CATI	Yes	Point prevalence	Spain: 12.0%, Ireland 13.0%, UK: 13.0%, France 15.0%, Switzerland 16.0%, Denmark 16.0%, Germany 17.0%,	5

	Author	Year	Country	Sample size	Sex distribution	Age	Education	Participant recruitment	Region	Study design	Diagnostic criteria	Diagnostic instrument	Clinical interview	Type of prevalence measure	Prevalence estimate	JBI
			Poland, Norway	Sweden: 2,563, Finland: 2,004, Austria: 2,004, Belgium: 2,451, Italy: 3,849, Poland: 3,812, Norway: 2,018											Netherlands 18.0%, Sweden 18.0%, Finland 19.0%, Austria 21.0%, Belgium 23.0%, Italy 26.0%, Poland 27.0%, Norway 30.0%	
**10.**	Fröhlich et al. [12]	2006	Germany	4,181	Males: 50.3%,	18 – 65	Educational Distributi	Population registries	Diverse	Cross-sectional	DSM-IV	M-CIDI	Yes	12-month prevalence	8.1%	8

	Author	Year	Country	Sample size	Sex distribution	Age	Education	Participant recruitment	Region	Study design	Diagnostic criteria	Diagnostic instrument	Clinical interview	Type of prevalence measure	Prevalence estimate	JBI
					Females: 49.7%		on (Social Class Index): Low: 19.1% Medium: 57.6% High: 23.3%	, Stratified random sample								
**11.**	Jablonska et al. [15]	2006	Sweden	3,616	Only females	18 – 64	High (41.2%), Intermediate (37.3%), Low (21.5%)	Random sample	Urban	Cross-sectional	Definition: 3 months	The Pain Questionnaire	No	Point prevalence	40.0	6

	Author	Year	Country	Sample size	Sex distribution	Age	Education	Participant recruitment	Region	Study design	Diagnostic criteria	Diagnostic instrument	Clinical interview	Type of prevalence measure	Prevalence estimate	JBI
**12.**	Svebak et al. [22]	2006	Norway	64,690	Males: 45.5%, Females: 54.5%	≥20	N/A	National registry	Urban	Longitudinal	Definition: 3 months	Standardized Nordic Questionnaire	No	12-month prevalence	44.6%	8
**13.**	Wijnhoven et al. [23]	2006	The Netherlands	3,664	Males 41%, Females 52%	25-64	N/A	random sampling method	Both	Longitudinal cohort study	Definition 3 months	Self-developed	No	Point prevalence	Males: 39%, Females 45%	8
**14.**	Bouhassira et al. [5]	2008	France	23,712	Males 46%, Females 54%	≥18	N/A	Polls registry	Diverse	Cross-sectional	Definition: 3 months	Brief Pain Inventory, NRS	No	Point prevalence	31.7%; (95%CI: 31.1–32.3)	8
**15.**	Gunnarsdottir et al. [14]	2010	Iceland	1,286	Males 44%, Females 56%	≥18		National registry	Both	Cross-sectional	Definition 3 months	Brief Pain Inventory	No	Point prevalence	30.6%	7
**16.**	Landmark et al. [18]	2012	Norway	94,194	Males 45.3%,	≥20	Years of education	National registry	Urban	Longitudinal	Definition: 6 months	Self-developed, SF-8	No	12-month prevalence	28% (95% CI	8

	Author	Year	Country	Sample size	Sex distribution	Age	Education	Participant recruitment	Region	Study design	Diagnostic criteria	Diagnostic instrument	Clinical interview	Type of prevalence measure	Prevalence estimate	JBI
					Females 54.7%		Women: ≤10: 13.1% 11–13: 44.4% ≥14: 42.5%<br><br>Men: ≤10: 10.0% 11–13: 55.3% ≥14: 34.7%								27 to 30)	
**17.**	Azevedo et al. [2]	2012	Portugal	5,094	Male: 47.7%, Female: 52.3%	≥18	Education:<br><br>More than 12	Telephone survey, random sample	Urban	Cross-sectional	IASP	Self-developed	Yes	Point prevalence	36.7% (95%[35.3-38.2]	9

	Author	Year	Country	Sample size	Sex distribution	Age	Education	Participant recruitment	Region	Study design	Diagnostic criteria	Diagnostic instrument	Clinical interview	Type of prevalence measure	Prevalence estimate	JBI
							years (higher education): 19.9%<br>10–12 years (secondary education): 27.8%<br>5–9 years (basic 2nd and 3rd cycles): 20.3%<br>1–4 years (basic 1st cycle): 25.9%									

	Author	Year	Country	Sample size	Sex distribution	Age	Education	Participant recruitment	Region	Study design	Diagnostic criteria	Diagnostic instrument	Clinical interview	Type of prevalence measure	Prevalence estimate	JBI
							No education: 3.3%									
**18.**	Björnsdóttir et al. [4]	2013	Iceland	5906	Males 46.1%, Females 53.9%	18–79	N/A	Stratified random sampling	Diverse	Cross-sectional	IASP	Self-developed, SF-36, IPAQ,	No	Point prevalence	19.9%	8
**19.**	Dueñas et al. [11]	2015	Spain	1,957	Males 49.62%, Females 50.38%	≥18	No Education: 19.2% Primary Education: 26.4% Secondary Education: 25.5% Vocational	Telephone survey, Random sample	Diverse	Cross-sectional	IASP	Self-developed	Yes	Point prevalence	16.6% (95%: 14.9–18.3)	8

	Author	Year	Country	Sample size	Sex distribution	Age	Education	Participant recruitment	Region	Study design	Diagnostic criteria	Diagnostic instrument	Clinical interview	Type of prevalence measure	Prevalence estimate	JBI
							Training: 12.6% University Education: 16.3%									
**20.**	MacFarlane et al. [19]	2015	UK	503,325	Males 45.8%, Females 54.2%	40–69		Health service registry	Both	Cross-sectional	Definition: 6 months		No	Point prevalence	42.9%	6
**21.**	Mundal et al. [20]	2016	Norway	78,973	Males 43%, Females 57%	≥ 20	Years of education<br><br>Women:<br>≤10: 13.1%<br>11–13: 44.4%<br>≥14: 42.5%	Birth registration	Urban	Longitudinal	Definition 3 months using Standardized Nordic Questionnair	SNQ	Yes	12 month prevalence	48%	9

	Author	Year	Country	Sample size	Sex distribution	Age	Education	Participant recruitment	Region	Study design	Diagnostic criteria	Diagnostic instrument	Clinical interview	Type of prevalence measure	Prevalence estimate	JBI
							Men: ≤10: 10.0% 11–13: 55.3% ≥14: 34.7%				e (SNQ)					
**22.**	Del Giorno et al. [10]	2017	Italy	1,293	Males 42.1%, Females 57.2%	≥18	N/A	National registry	Diverse	Cross-sectional	Definition: 3 months	Self-developed	No	Point prevalence	28.4%	5
**23.**	Landmark et al. [17]	2018	Norway	3,405	Males 45.3%, Females 54.7%	≥ 20	Years of education Women: ≤10: 13.1% 11–13: 44.4%	National registry	Both	Longitudinal	Definition: 6 months	Self-developed, SF-8	No	Point prevalence	30.0%	8

	Author	Year	Country	Sample size	Sex distribution	Age	Education	Participant recruitment	Region	Study design	Diagnostic criteria	Diagnostic instrument	Clinical interview	Type of prevalence measure	Prevalence estimate	JBI
							≥14: 42.5%<br><br>Men:<br>≤10: 10.0%<br>11–13: 55.3%<br>≥14: 34.7%									

3.2.3 Pubmed Search strategy

Group 1	((((((((((((((((persistent pain) OR (chronic pain)) OR (Fibromy*)) OR (tension headache*)) OR ("chronic musculoskeletal pain")) OR ("functional abdominal pain")) OR ("tension type headache")) OR (fibrositis)) OR (fibromyositis)) OR (myofibrositis)) OR ("chronic widespread pain")) OR ("widespread musculoskeletal pain")) OR ("myofascial pain")) OR ("chronic intractable benign pain*")) OR (non cardiac chest pain)) OR (non specific chest pain)) OR (atypical pain)
Group 2	(Prevalence*) OR (Epidemiol*)
Results:	#1 AND #2

3.2.4 Web of Science Search strategy

Group 1	(((((((((((((((ALL=(persistent pain)) OR ALL=(chronic pain)) OR ALL=(Fibromy*)) OR ALL=(tension headache*)) OR ALL=("chronic musculoskeletal pain")) OR ALL=("functional abdominal pain")) OR ALL=("tension type headache")) OR ALL=(fibrositis)) OR ALL=(fibromyositis)) OR ALL=(myofibrositis)) OR ALL=("chronic widespread pain")) OR ALL=("widespread musculoskeletal pain")) OR ALL=("myofascial pain")) OR ALL=("chronic intractable benign pain*")) OR ALL=("non cardiac chest pain")) OR ALL=("non specific chest pain")) OR ALL=("atypical pain")
Group 2	(ALL=(Prevalence*)) OR ALL=(Epidemiol*)
Results:	#1 AND #2

3.2.5 Embase Search strategy

('persistent pain'/exp OR 'chronic pain'/exp OR 'fibromyalgia'/exp OR 'tension headache'/exp OR 'chronic musculoskeletal pain'/exp OR 'functional abdominal pain'/exp OR 'tension type headache'/exp OR 'fibrositis'/exp OR 'fibromyositis'/exp OR 'chronic widespread pain'/exp OR 'myofascial pain'/exp OR 'chronic intractable benign pain' OR 'non cardiac chest pain'/exp OR 'persistent pain' OR 'chronic pain' OR fibromy* OR 'tension headache*' OR 'chronic musculoskeletal pain' OR 'functional abdominal pain' OR 'tension type headache' OR fibrositis OR fibromyositis OR myofibrositis OR 'chronic widespread pain' OR 'widespread musculoskeletal pain' OR 'myofascial pain' OR 'chronic intractable benign pain*' OR 'non cardiac chest pain' OR 'non specific chest pain' OR 'atypical pain') AND ('prevalence'/exp OR 'epidemiology'/exp OR prevalence* OR epidemiol*)

3.2.6 Search strategy Cochrane library

(persistent NEXT pain) OR

(chronic NEXT pain) OR

(Fibromy*) OR

(tension NEXT headache*) OR

(chronic NEXT musculoskeletal NEXT pain) OR

(functional NEXT abdominal NEXT pain) OR

(tension NEXT type NEXT headache) OR

(fibrositis) OR

(fibromyositis) OR

(myofibrositis) OR

(chronic NEXT widespread NEXT pain) OR

(widespread NEXT musculoskeletal NEXT pain) OR

(myofascial NEXT pain) OR

(chronic NEXT intractable NEXT benign NEXT pain*) OR

(non NEXT cardiac NEXT chest NEXT pain) OR

(non NEXT specific NEXT chest NEXT pain) OR

(atypical NEXT pain) AND

(Prevalence*) OR

(Epidemiol*)

3.2.7 Funnel plot

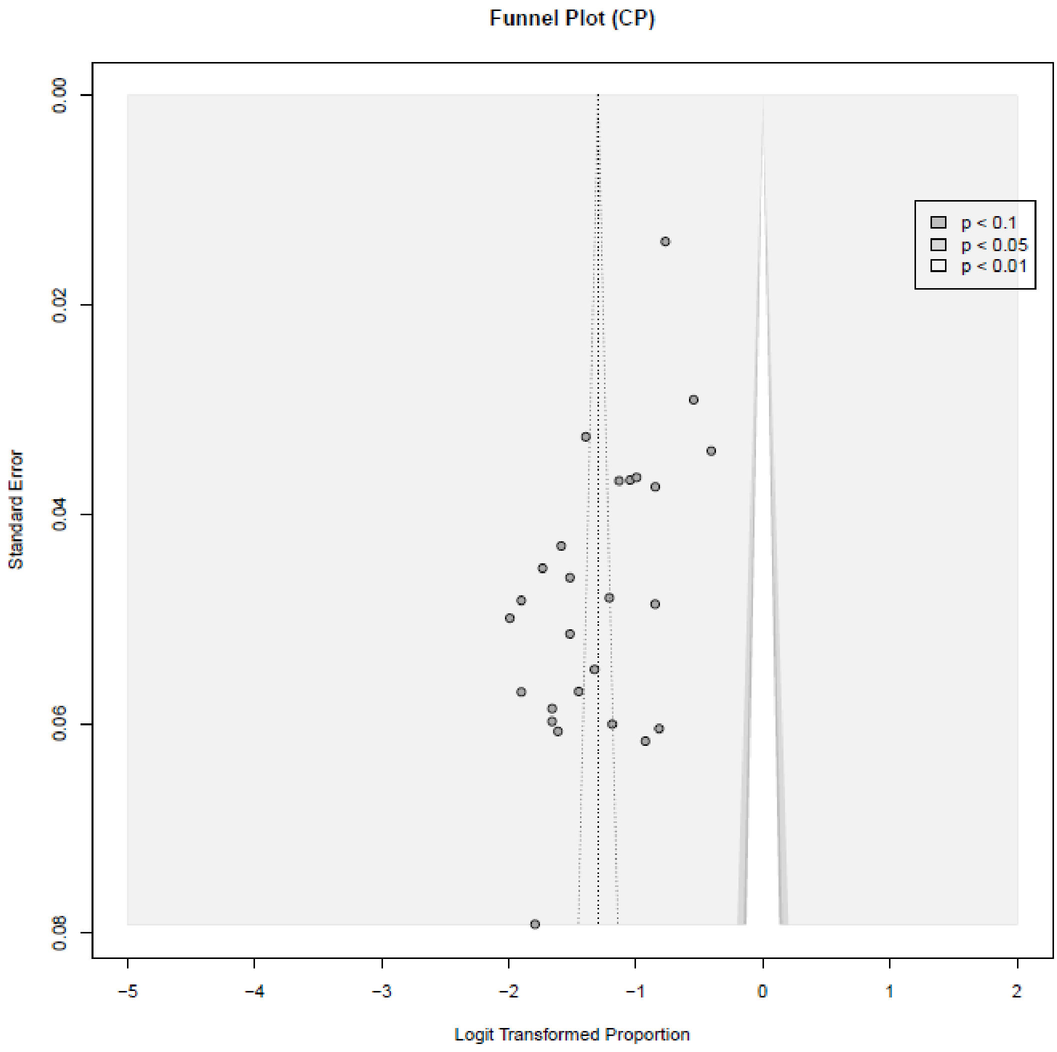

Figure S1. Funnel plot of the included studies on the point prevalence of chronic pain. Significant asymmetry can be detected.

References

[1] Andersson HI. The epidemiology of chronic pain in a Swedish rural area. Qual Life Res 1994;3(1):S19-S26.

[2] Azevedo LF, Costa-Pereira A, Mendonça L, Dias CC, Castro-Lopes JM. Epidemiology of chronic pain: a population-based nationwide study on its prevalence, characteristics and associated disability in Portugal. J Pain 2012;13(8):773-783.

[3] Bergman S, Herrström P, Högström K, Petersson IF, Svensson B, Jacobsson LT. Chronic musculoskeletal pain, prevalence rates, and sociodemographic associations in a Swedish population study. J Rheumatol 2001;28(6):1369-1377.

[4] Björnsdóttir S, Jónsson S, Valdimarsdóttir U. Functional limitations and physical symptoms of individuals with chronic pain. Scand J Rheumatol 2013;42(1):59-70.

[5] Bouhassira D, Lantéri-Minet M, Attal N, Laurent B, Touboul C. Prevalence of chronic pain with neuropathic characteristics in the general population. Pain 2008;136(3):380-387.

[6] Brattberg G, Thorslund M, Wikman A. The prevalence of pain in a general population. The results of a postal survey in a county of Sweden. Pain 1989;37(2):215-222.

[7] Breivik H, Collett B, Ventafridda V, Cohen R, Gallacher D. Survey of chronic pain in Europe: prevalence, impact on daily life, and treatment. Eur J Pain 2006;10(4):287-333.

[8] Catala E, Reig E, Artes M, Aliaga L, López J, Segu J. Prevalence of pain in the Spanish population: telephone survey in 5000 homes. Eur J Pain 2002;6(2):133-140.

[9] Chrubasik S, Junck H, Zappe H, Stutzke O. A survey on pain complaints and health care utilization in a German population sample. Eur J Anaesthesiol 1998;15(4):397-408.

[10] Del Giorno R, Frumento P, Varrassi G, Paladini A, Coaccioli S. Assessment of chronic pain and access to pain therapy: a cross-sectional population-based study. J Pain Res 2017;10:2577.

[11] Dueñas M, Salazar A, Ojeda B, Fernández-Palacín F, Micó JA, Torres LM, Failde I. A nationwide study of chronic pain prevalence in the general Spanish population: identifying clinical subgroups through cluster analysis. Pain Med 2015;16(4):811-822.

[12] Fröhlich C, Jacobi F, Wittchen H-U. DSM–IV pain disorder in the general population. Eur Arch Psychiatry Clin Neurosci 2006;256(3):187-196.

[13] Grabe HJ, Meyer C, Hapke U, Rumpf H-J, Freyberger HJ, Dilling H, John U. Somatoform pain disorder in the general population. Psychother Psychosom 2003;72(2):88-94.

[14] Gunnarsdottir S, Ward SE, Serlin RC. A population based study of the prevalence of pain in Iceland. Scand J Pain 2010;1(3):151-157.

[15] Jablonska B, Soares JJ, Sundin Ö. Pain among women: associations with socio-economic and work conditions. Eur J Pain 2006;10(5):435-447.

[16] Jacobi F, Wittchen H-U, Hölting C, Höfler M, Pfister H, Müller N, Lieb R. Prevalence, co-morbidity and correlates of mental disorders in the general population: results from the German Health Interview and Examination Survey (GHS). Psychol Med 2004;34(4):597-611.

[17] Landmark T, Dale O, Romundstad P, Woodhouse A, Kaasa S, Borchgrevink PC. Development and course of chronic pain over 4 years in the general population: The HUNT pain study. Eur J Pain 2018;22(9):1606-1616.

[18] Landmark T, Romundstad P, Dale O, Borchgrevink PC, Kaasa S. Estimating the prevalence of chronic pain: validation of recall against longitudinal reporting (the HUNT pain study). Pain 2012;153(7):1368-1373.

[19] Macfarlane GJ, Beasley M, Smith BH, Jones GT, Macfarlane TV. Can large surveys conducted on highly selected populations provide valid information on the epidemiology of common health conditions? An analysis of UK Biobank data on musculoskeletal pain. Br J Pain 2015;9(4):203-212.

[20] Mundal I, Bjørngaard JH, Nilsen TI, Nicholl BI, Gråwe RW, Fors EA. Long-term changes in musculoskeletal pain sites in the general population: the HUNT study. J Pain 2016;17(11):1246-1256.

[21] Rustøen T, Wahl AK, Hanestad BR, Lerdal A, Paul S, Miaskowski C. Prevalence and characteristics of chronic pain in the general Norwegian population. Eur J Pain 2004;8(6):555-565.

[22] Svebak S, Hagen K, Zwart J-A. One-year prevalence of chronic musculoskeletal pain in a large adult Norwegian county population: Relations with age and gender–The HUNT Study. J Musculoskelet Pain 2006;14(1):21-28.

[23] Wijnhoven HA, De Vet HC, Picavet HSJ. Prevalence of musculoskeletal disorders is systematically higher in women than in men. Clin J Pain 2006;22(8):717-724.
